# Incorporating medical code descriptions for diagnosis prediction in healthcare

**DOI:** 10.1186/s12911-019-0961-2

**Published:** 2019-12-19

**Authors:** Fenglong Ma, Yaqing Wang, Houping Xiao, Ye Yuan, Radha Chitta, Jing Zhou, Jing Gao

**Affiliations:** 1 Pennsylvania State University, State College, PA USA; 2 University at Buffalo, Buffalo, NY USA; 3 Georgia State University, Atlanta, GA USA; 4 Beijing University of Technology, Beijing, China; 5 Kira Systems, Toronto, ON Canada; 6 eHealth Inc., Mountain View, CA USA

**Keywords:** Healthcare informatics, Diagnosis prediction, Medical code embeddings

## Abstract

**Background:**

Diagnosis aims to predict the future health status of patients according to their historical electronic health records (EHR), which is an important yet challenging task in healthcare informatics. Existing diagnosis prediction approaches mainly employ recurrent neural networks (RNN) with attention mechanisms to make predictions. However, these approaches ignore the importance of code descriptions, i.e., the medical definitions of diagnosis codes. We believe that taking diagnosis code descriptions into account can help the state-of-the-art models not only to learn meaning code representations, but also to improve the predictive performance, especially when the EHR data are insufficient.

**Methods:**

We propose a simple, but general diagnosis prediction framework, which includes two basic components: diagnosis code embedding and predictive model. To learn the interpretable code embeddings, we apply convolutional neural networks (CNN) to model medical descriptions of diagnosis codes extracted from online medical websites. The learned medical embedding matrix is used to embed the input visits into vector representations, which are fed into the predictive models. Any existing diagnosis prediction approach (referred to as the base model) can be cast into the proposed framework as the predictive model (called the enhanced model).

**Results:**

We conduct experiments on two real medical datasets: the MIMIC-III dataset and the Heart Failure claim dataset. Experimental results show that the enhanced diagnosis prediction approaches significantly improve the prediction performance. Moreover, we validate the effectiveness of the proposed framework with insufficient EHR data. Finally, we visualize the learned medical code embeddings to show the interpretability of the proposed framework.

**Conclusions:**

Given the historical visit records of a patient, the proposed framework is able to predict the next visit information by incorporating medical code descriptions.

## Background

The immense accumulation of Electronic Healthcare Records (EHR) makes it possible to directly predict patients’ future health status, which is done by analyzing their historical visit records [1–4]. *Diagnosis prediction* attracts considerable attention from both healthcare providers and researchers. It aims to predict the diagnosis information of patients in the following visits. There are two key challenges for diagnosis prediction task as follows: (1) designing an accurate and robust predictive model to handle the temporal, high dimensional and noisy EHR data; and (2) reasonably interpreting the advantages and effectiveness of the proposed models to both doctors and patients.

To address these challenges of diagnosis prediction task, many recurrent neural networks (RNN) based models [2–4] have been proposed. RETAIN [4] uses two recurrent neural networks with attention mechanisms to model the reverse time ordered EHR sequences. By employing a bidirectional recurrent neural network (BRNN), Dipole [2] enhances the prediction accuracy with different attention mechanisms. In order to guarantee the predictive performance, training the above mentioned models usually requires a lot of EHR data. However, there is a common problem for EHR data that is always existing medical codes of rare diseases. Those diagnosis codes infrequently appear in the EHR data. GRAM [3] has been proposed to overcome this issue. GRAM learns medical code representations by exploiting medical ontology information and the graph-based attention mechanism. For the rare medical codes, GRAM can alleviate the difficulties of learning their embeddings by considering their ancestors’ embeddings to guarantee the predictive performance. However, the performance of GRAM heavily depends on the choice of medical ontology. Thus, without specific input constraints, how to learn robust embeddings for medical codes is still the major challenge for accurate diagnosis prediction.

To resolve this challenge, we consider the “nature” of diagnosis codes, i.e., their medical descriptions. Actually, each diagnosis code has a formal description, which can be easily obtained from the Internet, such as Wikipedia or online medical websites. For example, the description of diagnosis code “428.32” is “*Chronic diastolic heart failure*” (http://www.icd9data.com/2015/Volume1/390-459/420-429/428/428.32.htm), and “*Rheumatic heart failure (congestive)*” is the description of diagnosis code “398.91” (http://www.icd9data.com/2015/Volume1/390-459/393-398/398/398.91.htm). Without considering the medical meanings of diagnosis codes, they are treated as two independent diseases in the EHR dataset. However, they both describe the same disease, i.e., “heart failure”. Thus, we strongly believe that **incorporating the descriptions of diagnosis codes** in the prediction should help the predictive models to improve the prediction accuracy and provide interpretable representations of medical codes, especially when the EHR data are insufficient.

The other benefit of incorporating diagnosis code descriptions is that it enables us to design a **general diagnosis prediction framework**. The input data of all the existing diagnosis prediction approaches are the same, i.e., a sequence of time-ordered visits, and each visit consists of some diagnosis codes. Thus, all the existing approaches, including, but not limited to RETAIN, Dipole and GRAM, can be extended to incorporate the descriptions of diagnosis codes to further improve their predictive performance.

In this paper, we propose a novel framework for diagnosis prediction task. It should be noted that all of the state-of-the-art diagnosis prediction approaches (referred to as *base models*) can be cast into the proposed framework. These base models enhanced by the proposed framework are thus called *enhanced models*. Specifically, the proposed framework consists of two components: diagnosis code embedding and predictive model. The diagnosis code embedding component aims to learn the medical representations of diagnosis codes according to their descriptions. In particular, for each word in the description, we obtain the pretrained vector representation from fastText [5]. Then the concatenation of all the words in each diagnosis code description is fed into a convolutional neural network (CNN) to generate the medical embeddings. Based on the learned medical embeddings of diagnosis codes, the predictive model component makes prediction. It first embeds the input visit information into a visit-level vector representation with the code embeddings, and then feeds this vector into the predictive model, which can be any existing diagnosis prediction approach.

We use two real medical datasets to illustrate the superior ability of the proposed framework on the diagnosis prediction task compared with several state-of-the-art approaches. Quantitative analysis is also conducted to validate the effectiveness of the proposed approaches with insufficient EHR data. Finally, we qualitatively analyze the interpretability of the enhanced approaches by visualizing the learned medical code embeddings against the embeddings learned by existing approaches. To sum up, we achieve the following contributions in this paper:

- We realize the importance of obtaining diagnosis code embeddings from their descriptions which can be directly extracted from the Internet.
- We propose a simple, but general and effective diagnosis prediction framework, which learns representations of diagnosis codes directly from their descriptions.
- All the state-of-the-art approaches can be cast into the proposed framework to improve the performance of diagnosis prediction.
- Experimental results on two medical datasets validate the effectiveness of the proposed framework and the interpretability for prediction results.

## Related Work

In this section, we briefly survey the work related to diagnosis prediction task. We first provide a general introduction about mining healthcare related data with deep learning techniques, and then survey the work of diagnosis prediction.

### Deep Learning for EHR

Several machine learning approaches are proposed to mine medical knowledge from EHR data [1, 6–10]. Among them, deep learning-based models have achieved better performance compared with traditional machine learning approaches [11–13]. To detect the characteristic patterns of physiology in clinical time series data, stacked denoising autoencoders (SDA) are used in [14]. Convolutional neural networks (CNN) are applied to predict unplanned readmission [15], sleep stages [16], diseases [17, 18] and risk [19–21] with EHR data. To capture the temporal characteristics of healthcare related data, recurrent neural networks (RNN) are widely used for modeling disease progression [22, 23], mining time series healthcare data with missing values [24, 25], and diagnosis classification [26] and prediction [2–4, 27].

### Diagnosis Prediction

Diagnosis prediction is one of the core research tasks in EHR data mining, which aims to predict the future visit information according to the historical visit records. Med2Vec [28] is the first unsupervised method to learn the interpretable embeddings of medical codes, but it ignores long-term dependencies of medical codes among visits. RETAIN [4] is the first interpretable model to mathematically calculate the contribution of each medical code to the current prediction by employing a reverse time attention mechanism in an RNN for binary prediction task. Dipole [2] is the first work to adopt bidirectional recurrent neural networks (BRNN) and different attention mechanisms to improve the prediction accuracy. GRAM [3] is the first work to apply graph-based attention mechanism on the given medical ontology to learn robust medical code embeddings even when lack of training data, and an RNN is used to model patient visits. KAME [29] uses high-level knowledge to improve the predictive performance, which is build upon GRAM.

However, different from all the aforementioned diagnosis prediction models, the proposed diagnosis prediction framework incorporates the descriptions of diagnosis codes to learn embeddings, which greatly improves the prediction accuracy and provide interpretable prediction results against the state-of-the-art approaches.

## Methods

In this section, we first mathematically define the notations used in the diagnosis prediction task, introduce preliminary concepts, and then describe the details of the proposed framework.

### Notations

We denote all the unique diagnosis codes from the EHR data as a code set $\mathcal {C} = \{c_{1}, c_{2}, \cdots, c_{|\mathcal {C}|}\}$, where $|\mathcal {C}|$ is the number of diagnosis codes. Let $|\mathcal {P}|$ denote the number of patients in the EHR data. For the *p*-th patient who has *T* visit records, the visiting information of this patient can be represented by a sequence of visits $\mathcal {V}^{(p)} = \left \{V_{1}^{(p)}, V_{2}^{(p)}, \cdots, V_{T}^{(p)}\right \}$. Each visit $V_{t}^{(p)}$ consists of multiple diagnosis codes, i.e., $V_{t}^{(p)} \subseteq \mathcal {C}$, which is denoted by a binary vector $\mathbf {x}_{t}^{(p)} \in \{0, 1\}^{|\mathcal {C}|}$. The *i*-th element of $\mathbf {x}_{t}^{(p)}$ is 1 if $V_{t}^{(p)}$ contains the diagnosis code *c*_*i*_. For simplicity, we drop the superscript (*p*) when it is unambiguous.

Each diagnosis code *c*_*i*_ has a formal medical description, which can be obtained from Wikipedia (https://en.wikipedia.org/wiki/List_of_ICD-9_codes) or ICD9Data.com (http://www.icd9data.com/). We denote all the unique words which are used to describe all the diagnosis codes as $\mathcal {W} = \{w_{1}, w_{2}, \cdots, w_{|\mathcal {W}|}\}$, and $c_{i}^{\prime } \subseteq \mathcal {W}$ as the description of *c*_*i*_, where $|\mathcal {W}|$ is the number of unique words.

With the aforementioned notations, the inputs of the proposed framework are the set of code descriptions $\left \{c_{1}^{\prime }, c_{2}^{\prime }, \cdots, c_{|\mathcal {C}|}^{\prime }\right \}$ and the set of time-ordered sequences of patient visits $\left \{\mathbf {x}_{1}^{(p)}, \mathbf {x}_{2}^{(p)}, \cdots, \mathbf {x}_{T-1}^{(p)}\right \}_{p=1}^{|\mathcal {P}|}$. For each timestep *t*, we aim to predict the information of the (*t*+1)-th visit. Thus, the outputs are $\left \{\mathbf {x}_{2}^{(p)}, \mathbf {x}_{3}^{(p)}, \cdots, \mathbf {x}_{T}^{(p)}\right \}_{p=1}^{|\mathcal {P}|}$.

### Preliminaries

In this subsection, we first introduce the commonly used techniques for modeling patients’ visits, and then list all the state-of-the-art diagnosis prediction approaches.

#### Fully Connected Layer

Deep learning based models are commonly used to model patients’ visits. Among existing models, fully connected layer (FC) is the simplest approach, which is defined as follows:

1 $$ \mathbf{h}_{t} = \mathbf{W}_{c} \mathbf{v}_{t} + \mathbf{b}_{c},  $$

where $\mathbf {v}_{t} \in \mathbb {R}^{d}$ is the input data, *d* is the input dimensionality, $\mathbf {W}_{c} \in \mathbb {R}^{|\mathcal {C}| \times d}$ and $\mathbf {b}_{c} \in \mathbb {R}^{|\mathcal {C}|}$ are the learnable parameters.

#### Recurrent Neural Networks

Recurrent Neural Networks (RNNs) have been shown to be effective in modeling healthcare data [2–4, 30]. Note that we use “RNN” to denote any Recurrent Neural Network variants, such as Long-Short Term Memory (LSTM) [31], T-LSTM [32] and Gated Recurrent Unit (GRU) [33]. In this paper, GRU is used to adaptively capture dependencies among patient visit information. GRU has two gates: One is the reset gate *r*, and the other is the update gate *z*. The reset gate *r* computes its state from both the new input and the previous memory. The function of *r* is to make the hidden layer drop irrelevant information. The update gate *z* controls how much information should be kept around from the previous hidden state. The mathematical formulation of GRU can be described as follows:

2 $$ \begin{aligned} \mathbf{z}_{t} & = \sigma(\mathbf{W}_{z} \mathbf{v}_{t} + \mathbf{U}_{z} \mathbf{h}_{t-1} + \mathbf{b}_{z}), \\ \mathbf{r}_{t} & = \sigma(\mathbf{W}_{r} \mathbf{\beta}_{t} + \mathbf{U}_{r} \mathbf{h}_{t-1} + \mathbf{b}_{r}),\\ \tilde{\mathbf{h}}_{t} & = \text{tanh}(\mathbf{W}_{h} \mathbf{\beta}_{t} + \mathbf{r}_{t} \circ \mathbf{U}_{h} \mathbf{h}_{t-1} + \mathbf{b}_{h}),\\ \mathbf{h}_{t} & = \mathbf{z}_{t} \circ \mathbf{h}_{t-1} + (\mathbf{1} - \mathbf{z}_{t}) \circ \tilde{\mathbf{h}}_{t}, \end{aligned}  $$

where $\mathbf {z}_{t} \in \mathbb {R}^{g}$ is the update gate at time *t*, *g* is the dimensionality of hidden states, *σ*() is the activation function, $\mathbf {h}_{t} \in \mathbb {R}^{g}$ is the hidden state, $\mathbf {r}_{t} \in \mathbb {R}^{g}$ is the reset gate at time *t*, $\tilde {\mathbf {h}}_{t} \in \mathbb {R}^{g}$ represents the intermediate memory, and ∘ denotes the element-wise multiplication. Matrices $\mathbf {W}_{z} \in \mathbb {R}^{g \times d}, \mathbf {W}_{r} \in \mathbb {R}^{g \times d}, \mathbf {W}_{h} \in \mathbb {R}^{g \times d}, \mathbf {U}_{z} \in \mathbb {R}^{g \times g}, \mathbf {U}_{r} \in \mathbb {R}^{g \times g}, \mathbf {U}_{h} \in \mathbb {R}^{g \times g}$ and vectors $\mathbf {b}_{z} \in \mathbb {R}^{g}, \mathbf {b}_{r} \in \mathbb {R}^{g}, \mathbf {b}_{h} \in \mathbb {R}^{g}$ are parameters to be learned. For simplicity, the GRU can be represented by

3 $$ \mathbf{h}_{t} = \text{GRU}(\mathbf{\beta}_{t}; \Omega),  $$

where *Ω* denotes all the parameters of GRU.

#### Attention Mechanisms

Attention mechanisms aim to distinguish the importance of different input data, and attention-based neural networks have been successfully used in diagnosis prediction task, including location-based attention [2, 4], general attention [2], concatenation-based attention [2], and graph-based attention [3]. In the following, we introduce two commonly used attention mechanisms: location-based and graph-based attention.

∙*Location-based Attention*. Location-based attention mechanism [2, 4] is to calculate the attention score for each visit, which solely depends on the current hidden state $\mathbf {h}_{i} \in \mathbb {R}^{g}$ (1≤*i*≤*t*) as follows:

4 $$ \alpha_{i} = \mathbf{W}_{\alpha}^{\top} \mathbf{h}_{i} + b_{\alpha},  $$

where $\mathbf {W}_{\alpha } \in \mathbb {R}^{g}$ and $b_{\alpha } \in \mathbb {R}$ are the parameters to be learned. According to Eq. (4), we can obtain an attention weight vector **α**=[*α*_1_,*α*_2_,⋯,*α*_*t*_] for the *t* visits. Then the softmax function is used to normalize **α**. Finally, we can obtain the context vector **c**_*t*_ according to the attention weight vector **α** and the hidden states from **h**_1_ to **h**_*t*_ as follows:

5 $$ \mathbf{c}_{t} = \sum_{i = 1}^{t} \alpha_{i} \mathbf{h}_{i}.  $$

We can observe that the context vector **c**_*t*_ is the weighted sum of all the visit information from time 1 to *t*.

∙*Graph-based Attention*. Graph-based attention [3] is proposed to learn robust representations of diagnosis codes even when the data volume is constrained, which explicitly employs the *parent-child* relationship among diagnosis codes with the given medical ontology to learn code embeddings.

Given a medical ontology $\mathcal {G}$ which is a directed acyclic graph (DAG), each leaf node of $\mathcal {G}$ is a diagnosis code *c*_*i*_ and each non-leaf node belongs to the set $\hat {\mathcal {C}}$. Each leaf node has a basic learnable embedding vector $\mathbf {e}_{i} \in \mathbb {R}^{d}$ ($1 \leq i \leq |\mathcal {C}|$), while $\mathbf {e}_{|\mathcal {C}| + 1}, \cdots, \mathbf {e}_{|\mathcal {C}| + |\hat {\mathcal {C}}|}$ represent the basic embeddings of the internal nodes $c_{|\mathcal {C}| + 1}, \cdots, c_{|\mathcal {C}| + |\hat {\mathcal {C}}|}$. Let $\mathcal {A}(i)$ be the node set of *c*_*i*_ and its ancestors, then the final embedding of diagnosis code *c*_*i*_ denoted by $\mathbf {g}_{i} \in \mathbb {R}^{d}$ can be obtained as follows:

6 $$ \mathbf{g}_{i} = \sum_{j \in \mathcal{A}(i)} \alpha_{ij} \mathbf{e}_{j}, \quad \sum_{j \in \mathcal{A}(i)} \alpha_{ij} = 1,  $$

where

7 $$ \alpha_{ij} = \frac{\exp(\theta(\mathbf{e}_{i}, \mathbf{e}_{j}))}{\sum_{k \in \mathcal{A}(i)} \exp(\theta(\mathbf{e}_{i}, \mathbf{e}_{k}))}.  $$

*θ*(·,·) is a scalar value and defined as

8 $$ \theta(\mathbf{e}_{i}, \mathbf{e}_{j}) = \mathbf{u}_{a}^{\top}\text{tanh}\left(\mathbf{W}_{a} \left[ \begin{array}{c} \mathbf{e}_{i} \\ \mathbf{e}_{j}\\ \end{array} \right] + \mathbf{b}_{a}\right),  $$

where $\mathbf {u}_{a} \in \mathbb {R}^{l}, \mathbf {W}_{a} \in \mathbb {R}^{l \times 2d}$ and $\mathbf {b}_{a} \in \mathbb {R}^{l}$ are parameters to be learned. Finally, graph-based attention mechanism generates the medical code embeddings $\mathbf {G} = \{\mathbf {g}_{1}, \mathbf {g}_{2}, \cdots, \mathbf {g}_{|\mathcal {C}|}\} \in \mathbb {R}^{d \times |\mathcal {C}|}$.

#### Base Models

Since the proposed framework is general, all the existing diagnosis prediction approaches can be cast into this framework and treated as base models. Table 1 shows the summary of all the state-of-the-art approaches with the aforementioned techniques. The detailed implementation of these base models is introduced in “Experimental Setup” section.

**Table 1:** Base models for diagnosis prediction

Base model	Visit modeling		Attention mechanism	
	FC	GRU	Location	Graph
MLP	*√*			
RNN [2–4]		*√*		
RNN _*a*_ [2]		*√*	*√*	
Dipole [2]		*√*	*√*	
RETAIN [4]		*√*	*√*	
GRAM [3]		*√*		*√*

### The Proposed Framework

Different from graph-based attention mechanism which specifies the relationships of diagnosis codes with the given medical ontology, we aim to learn the diagnosis code embeddings directly from their medical descriptions. The main components of the proposed diagnosis prediction framework are *diagnosis code embedding* and *predictive model*. Diagnosis code embedding component is to learn the medical embeddings with code descriptions, which can embed the visit information into a vector representation. Predictive model component aims to predict the future visit information according to the embedded visit representations. Obviously, the proposed framework can be trained end-to-end. Next, we provide the details of these two components.

#### Diagnosis Code Embedding

To embed the description of each diagnosis code into a vector representation, Convolutional Neural Networks (CNN) [34] can be employed. The benefit of applying CNN is to utilize layers with convolving filters to extract local features, which has shown its superior ability for natural language processing tasks, such as sentence modeling [35] and sentence classification [36].

**Fig. 1:**
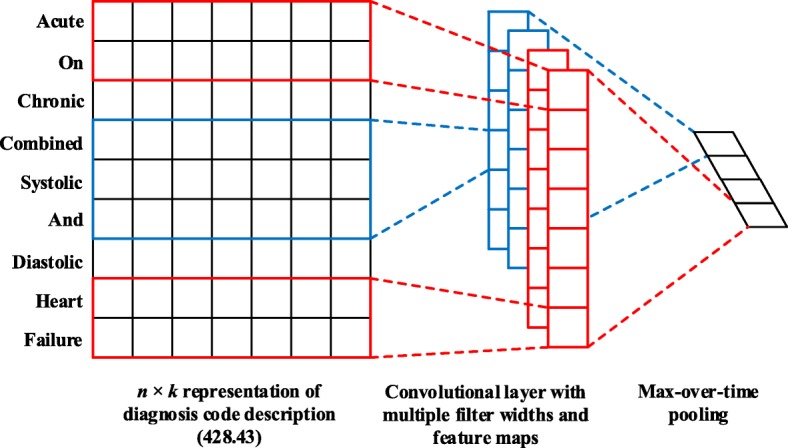
An Example of CNN Architecture for Diagnosis Code Embedding. The word window sizes are 2 (red line) and 3 (blue line) respectively, i.e., *q*=2. For each word window, there are 2 filters in the example, i.e., *m*=2. The dimensionality of this code embedding is 4, i.e., *d*=*mq*=4

Figure 1 shows the variant of the CNN architecture to embed each diagnosis code description $c_{i}^{\prime }$ into a vector representation **e**_*i*_. We first obtain the pre-trained embedding of each word *w*_*j*_ denoted as $\mathbf {l}_{j} \in \mathbb {R}^{k}$ from fastText [5], where *k* is the dimensionality. The description $c_{i}^{\prime }$ with length *n* (padded where necessary) is represented as

9 $$ \mathbf{l}_{1:n} = \mathbf{l}_{1} \oplus \mathbf{l}_{2} \oplus \cdots \oplus \mathbf{l}_{n},  $$

where ⊕ is the concatenation operator. Let *h* denote the size of a word window, and then **l**_*i*:*i*+*h*−1_ represents the concatenation of *h* words from **l**_*i*_ to **l**_*i*+*h*−1_. A filter $\mathbf {W}_{f} \in \mathbb {R}^{h \times k}$ is applied on the window of *h* words to produce a new feature $f_{i} \in \mathbb {R}$ with the ReLU activation function as follows:

10 $$ f_{i} = \text{ReLU}(\mathbf{W}_{f} \mathbf{l}_{i:i+h-1} + b_{f}),  $$

where $b_{f} \in \mathbb {R}$ is a bias term, and ReLU(*f*)= max(*f*,0). This filter is applied to each possible window of words in the whole description {**l**_1:*h*_,**l**_2:*h*+1_,⋯,**l**_*n*−*h*+1:*n*_} to generate a feature map $\mathbf {f} \in \mathbb {R}^{n-h+1}$ as follows:

11 $$ \mathbf{f} = [f_{1}, f_{2}, \cdots, f_{n-h+1}].  $$

Next, max pooling technique [37] is used over the feature map to obtain the most important feature, i.e., $\hat {f} = \max (\mathbf {f})$. In this way, one filter produces one feature. To obtain multiple features, we use *m* filters with varying window sizes. Here, we use *q* to denote the number of different window sizes. All the extracted features are concatenated to represent the embedding of each diagnosis code $\mathbf {e}_{i} \in \mathbb {R}^{d}$ (*d*=*mq*). Finally, we can obtain the diagnosis code embedding matrix $\mathbf {E} \in \mathbb {R}^{d \times |\mathcal {C}|}$, where **e**_*i*_ is the *i*-th column of **E**.

The advantage of the proposed CNN-based diagnosis code embedding approach is that it easily makes the diagnosis codes with similar meanings obtain similar vector representations. Thus, for those diagnosis codes without sufficient training EHR data, they still can learn reasonable vector representations, which further helps the model to improve the predictive performance. In the following, we will introduce how to use the produced medical embeddings for the diagnosis prediction task.

#### Predictive Model

Based on the learned diagnosis code embedding matrix **E**, we can predict patients’ future visit information with a predictive model. Given a visit $\mathbf {x}_{t} \in \{0, 1\}^{|\mathcal {C}|}$, we first embed **x**_*t*_ into a vector representation $\mathbf {v}_{t} \in \mathbb {R}^{d}$ with **E** as follows:

12 $$ \mathbf{v}_{t} = \text{tanh}(\mathbf{E}\mathbf{x}_{t} + \mathbf{b}_{v}),  $$

where $\mathbf {b}_{v} \in \mathbb {R}^{d}$ is the bias vector to be learned. Then **v**_*t*_ is fed into the predictive model to predict the (*t*+1)-th visit information, i.e., $\hat {\mathbf {y}}_{t}$. Next, we cast state-of-the-art diagnosis prediction approaches into the proposed framework as the predictive models.

∙*Enhanced MLP* (MLP +). The simplest predictive model is only using a Multilayer Perceptron (MLP) with two layers: a fully-connected layer and a softmax layer, i.e.,

13 $$ \hat{\mathbf{y}}_{t} = \text{softmax}(\mathbf{h}_{t}),  $$

where **h**_*t*_ is obtained from Eq. (1). This model works well when both the number of diagnosis codes and patients’ visits are small. However, MLP + does not use historical visit information for the prediction. To overcome the shortage of MLP +, we employ Recurrent Neural Networks (RNN) to handle more complicated scenarios.

∙*Enhanced RNN* (RNN +). For RNN +, the visit embedding vector **v**_*t*_ is fed into a GRU, which produces a hidden state $\mathbf {h}_{t} \in \mathbb {R}^{g}$ as follows:

14 $$ \mathbf{h}_{t} = \text{GRU}(\mathbf{v}_{t}; \Omega).  $$

Then the hidden state **h**_*t*_ is fed through the softmax layer to predict the (*t*+1)-th visit information as follows:

15 $$ \hat{\mathbf{y}}_{t} = \text{softmax}(\mathbf{W}_{c} \mathbf{h}_{t} + \mathbf{b}_{c}),  $$

where $\mathbf {W}_{c} \in \mathbb {R}^{|\mathcal {C}| \times g}$. Note that RNN + only uses the *t*-th hidden state to make the prediction, which does not utilize the information of visits from time 1 to *t*−1. To consider all the information before the prediction, attention-based models are proposed in the following.

∙*Enhanced Attention-based RNN* (RNN _*a*_+). According to Eq. (14), we can obtain all the hidden states **h**_1_,**h**_2_,⋯,**h**_*t*_. Then location-based attention mechanism is applied to obtain the context vector **c**_*t*_ with Eq. (5). Finally, the context vector **c**_*t*_ is fed into the softmax layer to make predictions as follows:

16 $$ \hat{\mathbf{y}}_{t} = \text{softmax}(\mathbf{W}_{c} \mathbf{c}_{t} + \mathbf{b}_{c}).  $$

∙*Enhanced Dipole* (Dipole +). Actually, one drawback of RNN is that prediction performance will drop when the length of sequence is very large [38]. To overcome this drawback, Dipole [2] which uses bidirectional recurrent networks (BRNN) with attention mechanisms are proposed to improve the prediction performance.

Given the visit embeddings from **v**_1_ to **v**_*t*_, a BRNN can learn two sets of hidden states: forward hidden states $\overrightarrow {\mathbf {h}}_{1}, \cdots, \overrightarrow {\mathbf {h}}_{t}$ and backward hidden states $\overleftarrow {\mathbf {h}}_{1}, \cdots, \overleftarrow {\mathbf {h}}_{t}$. By concatenating $\overrightarrow {\mathbf {h}}_{t}$ and $\overleftarrow {\mathbf {h}}_{t}$, we can obtain the final hidden state $\mathbf {h}_{t} = [\overrightarrow {\mathbf {h}}_{t}; \overleftarrow {\mathbf {h}}_{t}]^{\top }$ ($\mathbf {h}_{t} \in \mathbb {R}^{2g}$). Then location-based attention mechanism is used to produce the context vector $\mathbf {c}_{t} \in \mathbb {R}^{2g}$ with Eq. (4) ($\mathbf {W}_{\alpha } \in \mathbb {R}^{2g}$). With the learned **c**_*t*_, Dipole + can predict the (*t*+1)-th visit information with a softmax layer, i.e., Eq. (16) with $\mathbf {W}_{c} \in \mathbb {R}^{|\mathcal {C}| \times 2g}$.

∙*Enhanced RETAIN* (RETAIN +). RETAIN [4] is an interpretable diagnosis prediction model, which uses two *reverse time-ordered* GRUs and attention mechanisms to calculate the contribution scores of all the appeared diagnosis codes before the prediction.

The visit-level attention scores can be obtained using Eq. (4). For the code-level attention scores, RETAIN employs the following function:

17 $$ \mathbf{\beta}_{t} = \text{tanh}(\mathbf{W}_{\beta} \mathbf{h}_{t} + \mathbf{b}_{\beta}),  $$

where $\mathbf {W}_{\beta } \in \mathbb {R}^{d \times g}$ and $\mathbf {b}_{\beta } \in \mathbb {R}^{d}$ are parameters. Then the context vector $\mathbf {c}_{t} \in \mathbb {R}^{d}$ is obtained as follows:

18 $$ \mathbf{c}_{t} = \sum_{i = 1}^{t} \alpha_{i} \mathbf{\beta}_{i} \circ \mathbf{v}_{i}.  $$

With the generated context vector **c**_*t*_ and Eq. (16) ($\mathbf {W}_{c} \in \mathbb {R}^{d}$), RETAIN + can predict the (*t*+1)-th patient’s health status.

∙*Enhanced GRAM* (GRAM +). GRAM [3] is the state-of-the-art approach to learn reasonable and robust representations of diagnosis codes with medical ontologies. To enhance GRAM with the proposed framework, instead of randomly assigning the basic embedding vectors $\mathbf {e}_{1}, \cdots, \mathbf {e}_{|\mathcal {C}|}$, we use diagnosis code descriptions to learn those embeddings, i.e., **E**. Note that the non-leaf nodes are still randomly assigned basic embeddings.

With the learned diagnosis code embedding matrix **G** as described in “Preliminaries” section, we can obtain visit-level embedding **v**_*t*_ with Eq. (12) (i.e., replacing **E** to **G**). Using Eqs. (14) and (15), GRAM + predicts the (*t*+1)-th visit information.

**Remark:** A key benefit of the proposed framework is its flexibility and transparency relative to all the existing diagnosis prediction models. Beyond all the aforementioned base approaches, more effective and complicated diagnosis prediction models can also be easily cast into the proposed framework.

## Results

In this section, we first introduce two real world medical datasets used in the experiments, and then describe the settings of experiments. Finally, we validate the proposed framework on the two datasets.

### Real-World Datasets

Two medical claim datasets are used in our experiments to validate the proposed framework, which are the MIMIC-III dataset [39] and the Heart Failure dataset.

∙ The MIMIC-III dataset, a publicly available EHR dataset, consists of medical records of 7,499 intensive care unit (ICU) patients over 11 years. For this dataset, we chose the patients who made at least two visits.

∙ The Heart Failure dataset is an insurance claim dataset, which has 4,925 patients and 341,865 visits from the year 2004 to 2015. The patient visits were grouped by week [2], and we chose patients who made at least two visits. Table 2 shows more details about the two datasets.

**Table 2:** Statistics of MIMIC-III and heart failure datasets

Dataset	MIMIC-III	Heart failure
# of patients	7,499	4,925
# of visits	19,911	341,865
Avg. visits per patient	2.66	69.41
# of unique ICD9 codes	4,880	6,747
Avg. # of diagnosis codes per visit	13.06	3.92
Max # of diagnosis codes per visit	39	54
# of words in code descriptions	2,800	3,397
# of category codes	171	149
Avg. # of category codes per visit	10.16	3.33
Max # of category codes per visit	30	33

Diagnosis prediction task aims to predict the diagnosis information of the next visit. In our experiments, we intend to predict the diagnosis categories as [2, 3], instead of predicting the real diagnosis codes. Predicting category information not only increases the training speed and predictive performance, but also guarantees the sufficient granularity of all the diagnoses. The nodes in the second hierarchy of the ICD9 codes are used as the category labels. For example, the category label of diagnosis code “428.43: Acute on chronic combined systolic and diastolic heart failure” is “Diseases of the circulatory system (390 −459)”.

### Experimental Setup

We first introduce the state-of-the-art diagnosis prediction approaches as base models, then describe the measures to evaluate the prediction results of all the approaches, and finally present the details of our experiment implementation.

#### Base Models

In our experiments, we use the following six approaches as base models:

∙MLP. MLP is a naive method, which first embeds the input visit **x**_*t*_ into a vector space **v**_*t*_, and then uses Eq. (1) and Eq. (13) to predict the (*t*+1)-th visit information.

∙RNN. RNN is a commonly used model. The input visit is first embedded into a visit-level representation **v**_*t*_ with a randomly initialized embedding matrix. Then **v**_*t*_ is fed into a GRU, and the GRU outputs the hidden state **h**_*t*_ (Eq. (14)), which is used to predict the next visit information with Eq. (15).

∙RNN_*a*_ [2]. RNN_*a*_ adds the location-based attention mechanism into RNN. After the GRU outputs the hidden states **h**_1_,**h**_2_,⋯,**h**_*t*_, RNN _*a*_ employs Eqs. (4) and (5) to calculate the context vector **c**_*t*_. Finally, RNN _*a*_ makes the predictions using the learned **c**_*t*_ and Eq. (16).

∙Dipole [2]. Dipole is the first work to apply bidirectional recurrent neural networks to diagnosis prediction task. In our experiments, we use location-based attention mechanism. Compared with RNN _*a*_, the difference is that Dipole uses two GRUs to generate the hidden states, and then concatenates these two sets of hidden states to calculate the context vector **c**_*t*_ with location-based attention mechanism. ∙RETAIN [4]. RETAIN focuses on interpreting the prediction results with a two-level attention model. RETAIN uses a reverse time-ordered visit sequence to calculate the visit-level attention score with Eq. (4). The other GRU is used to compute the code-level attention weight with Eq. (17). The context vector **c**_*t*_ is obtained using Eq. (18). Based on this context vector, RETAIN predicts the (*t*+1)-th diagnosis codes.

∙GRAM [3]. GRAM is the first work to employ medical ontologies to learn diagnosis code representations and predict the future visit information with recurrent neural networks. GRAM first learns the diagnosis code embedding matrix **G** with graph-based attention mechanism (Eq. (6)). With the learned **G**, the input visit **x**_*t*_ is embedded into a visit-level representation **v**_*t*_, which is fed into a GRU to produce the hidden state **h**_*t*_. Equation (15) is used to make the final predictions.

For all the base models, we all design the corresponding enhanced approaches for comparison.

#### Evaluation Measures

To fairly evaluate the performance of all the diagnosis prediction approaches, we validate the results from aspects: visit level and code level with the measures *precision @k* and *accuracy @k*.

∙*Visit-level precision @k* is defined as the correct diagnosis codes in top *k* divided by min(*k*,|**y**_*t*_|), where |**y**_*t*_| is the number of category labels in the (*t*+1)-th visit.

∙ Given a visit *V*_*t*_ which contains multiple category labels, if the target label is in the top *k* guesses, then we get 1 and 0 otherwise. Thus, *code-level accuracy @k* is defined by the number of correct label predictions divided by the total number of label predictions.

*Visit-level precision @k* is used to evaluate the coarse-grained performance, while *code-level accuracy @k* evaluates the fine-grained performance. For all the measures, the greater values, the better performance. In the experiments, we vary *k* from 5 to 30.

#### Implementation Details

We extract the diagnosis code descriptions from ICD9Data.com. All the approaches are implemented with Theano 0.9.0 [40]. We randomly divide the datasets into the training, validation and testing sets in a 0.75:0.10:0.15 ratio. The validation set is used to determine the best values of parameters in the 100 training iterations. For training models, we use Adadelta [41] with a min-batch of 100 patients. The regularization (*l*_2_ norm with the coefficient 0.001) is used for all the approaches.

In order to fairly compare the performance, we set the same *g*=128 (i.e., the dimensionality of hidden states) for all the base models and the enhanced approaches except MLP and MLP +. For the proposed approaches on both datasets, the size of word embeddings is 300, the word windows (*h*’s) are set as 2, 3 and 4, and thus *q*=3. For each word window, we use *m*=100 filters. For all the base models, we set *d*=180 on the MIMIC-III dataset and 150 on the Heart Failure dataset. For GRAM, *l* is 100.

### Results of Diagnosis Prediction

Table 3 shows the *visit-level precision* of all the base models and their corresponding enhanced approaches, and Table 4 lists the *code-level accuracy* with different *k*’s. From these two tables, we can observe that the enhanced diagnosis prediction approaches improve the prediction performance on both the MIMIC-III and Heart Failure datasets.

**Table 3:** The visit-level precision *@k* of diagnosis prediction task

Dataset	*@k*	MLP	MLP +	RNN	RNN +	RNN_*a*_	RNN_*a*_+	Dipole	Dipole +	RETAIN	RETAIN +	GRAM	GRAM +
MIMIC-III	5	0.6939	0.7124	0.6616	0.7160	0.6504	0.7083	0.6599	0.7074	0.6835	0.7167 ^∗^	0.6885	0.7132
	10	0.6441	0.6603	0.6145	0.6565	0.6021	0.6527	0.6116	0.6539	0.6361	0.6623 ^∗^	0.6424	0.6596
	15	0.6812	0.6926 ^∗^	0.6546	0.6906	0.6412	0.6856	0.6524	0.6903	0.6777	0.6918	0.6828	0.6918
	20	0.7420	0.7544 ^∗^	0.7199	0.7511	0.7109	0.7455	0.7159	0.7483	0.7403	0.7501	0.7434	0.7513
	25	0.7939	0.8070 ^∗^	0.7755	0.8019	0.7697	0.8009	0.7723	0.8020	0.7912	0.8010	0.7941	0.8028
	30	0.8357	0.8460	0.8186	0.8456	0.8142	0.8445	0.8169	0.8453	0.8335	0.8445	0.8377	0.8468 ^∗^
Heart failure	5	0.4451	0.4947	0.4890	0.5172	0.4976	0.5103	0.4964	0.5111	0.3751	0.5140	0.5341	0.5365 ^∗^
	10	0.6122	0.6206	0.6585	0.6879	0.6675	0.6817	0.6689	0.6829	0.5378	0.6828	0.7123	0.7159 ^∗^
	15	0.6996	0.7060	0.7436	0.7683	0.7496	0.7631	0.7514	0.7648	0.6372	0.7613	0.7901	0.7939 ^∗^
	20	0.7606	0.7643	0.8006	0.8213	0.8050	0.8174	0.8070	0.8167	0.7088	0.8143	0.8402	0.8442 ^∗^
	25	0.8100	0.8140	0.8425	0.8593	0.8453	0.8560	0.8476	0.8557	0.7655	0.8533	0.8761	0.8789 ^∗^
	30	0.8477	0.8511	0.8743	0.8879	0.8770	0.8857	0.8785	0.8846	0.8102	0.8826	0.9025	0.9047 ^∗^

^∗^ denotes the highest precision among all the approaches on the same *k*

**Table 4:** The code-level accuracy *@k* of diagnosis prediction task

Dataset	*@k*	MLP	MLP +	RNN	RNN +	RNN_*a*_	RNN_*a*_+	Dipole	Dipole +	RETAIN	RETAIN +	GRAM	GRAM +
MIMIC-III	5	0.3104	0.3181	0.2952	0.3193	0.2910	0.3162	0.2941	0.3155	0.3056	0.3198 ^∗^	0.3072	0.3183
	10	0.5040	0.5138	0.4796	0.5111	0.4693	0.5085	0.4767	0.5086	0.4980	0.5160 ^∗^	0.5003	0.5138
	15	0.6286	0.6352	0.6019	0.6335	0.5889	0.6290	0.5971	0.6325	0.6258	0.6360 ^∗^	0.6267	0.6348
	20	0.7114	0.7239 ^∗^	0.6894	0.7198	0.6822	0.7144	0.6845	0.7168	0.7129	0.7202	0.7130	0.7196
	25	0.7754	0.7852 ^∗^	0.7545	0.7804	0.7491	0.7785	0.7501	0.7795	0.7735	0.7806	0.7728	0.7794
	30	0.8214	0.8294 ^∗^	0.8040	0.8279	0.7987	0.8269	0.7990	0.8280	0.8198	0.8286	0.8220	0.8283
Heart failure	5	0.4580	0.5132	0.5599	0.5960	0.5699	0.5882	0.5687	0.5868	0.4085	0.5808	0.6152	0.6227 ^∗^
	10	0.6266	0.6412	0.6835	0.7169	0.6920	0.7109	0.6953	0.7105	0.5460	0.7042	0.7393	0.7455 ^∗^
	15	0.7124	0.7254	0.7603	0.7876	0.7645	0.7845	0.7702	0.7841	0.6512	0.7765	0.8088	0.8130 ^∗^
	20	0.7717	0.7827	0.8132	0.8355	0.8153	0.8334	0.8209	0.8307	0.7162	0.8261	0.8544	0.8580 ^∗^
	25	0.8206	0.8283	0.8516	0.8698	0.8532	0.8673	0.8580	0.8655	0.7684	0.8622	0.8872	0.8902 ^∗^
	30	0.8572	0.8635	0.8812	0.8958	0.8825	0.8943	0.8860	0.8923	0.8100	0.8899	0.9113	0.9134 ^∗^

^∗^ denotes the highest accuracy among all the approaches on the same *k*

#### Performance Analysis for the MIMIC-III Dataset

On the MIMIC-III dataset, the overall performance of all the enhanced diagnosis prediction approaches is better than that of all the base models. Among all the proposed approaches, RETAIN + and MLP + achieve higher accuracy. MLP + does not use recurrent neural networks and directly predicts the future diagnosis information with the learned visit embedding **v**_*t*_. RETAIN + utilizes the context vector which learns from visit-level and code-level attention scores, and the learned visit embeddings to make the final predictions. However, all the remaining proposed approaches use the hidden states outputted from GRUs to predict the next visit information. From the above analysis, we can conclude that directly adding visit embeddings into the final prediction can improve the predictive performance on the MIMIC-III dataset. This is reasonable because the average length of visits is small on the MIMIC-III dataset. The shorter visits may not help the RNN-based models to learn correct hidden states, and thus those methods can not achieve the highest accuracy.

This observation can also be found from the performance of all the base models. Compared with the naive base model MLP, the precision or accuracy of all the four RNN-based approaches is lower, including RNN, RNN _*a*_, Dipole and RETAIN. This again confirms that RNN-based models cannot work well with short sequences. Among all the RNN-based approaches, location-based attention models, RNN _*a*_ and Dipole, perform worse than RNN and RETAIN, which shows that learning attention mechanisms needs abundant EHR data. Compared with RNN, both the precision and accuracy of RETAIN are still higher. This demonstrates that directly using visit embedding in the final prediction may achieve better performance for the datasets with shorter visit sequences. GRAM can achieve comparable performance with the naive base model MLP. It proves that employing external information can compensate for the lack of training EHR data in diagnosis prediction task.

Here is an interesting observation: As expected, the performance improves as *k* increases, except the visit-level accuracy on the MIMIC-III dataset, due to the insufficiency of training data. Compared with the labels with abundant data, they obtain lower probabilities in the predictions. Thus, for the visits containing these labels without sufficient data, the number of correct predictions when *k* is 10 or 15 may be the same with that when *k*=5. However, they are divided by a bigger min(*k*,|**y**_*t*_|), which leads to the observation that the average performance is worse than that with *k*=5.

#### Performance Analysis for the Heart Failure Dataset

On the Heart Failure dataset, the enhanced approaches still perform better than the corresponding base models, especially GRAM + which achieves much higher accuracy than other approaches. The reason is that GRAM + not only uses medical ontologies to learn robust diagnosis code embeddings, but also employs code descriptions to further improve the performance, which can be validated from the comparison between the performance of GRAM and GRAM +.

Among all the approaches, both precision and accuracy of RETAIN are the lowest, which shows that directly using the visit-level embeddings in the final prediction may not work on the Heart Failure dataset, which can also be observed from the performance of MLP. However, taking code descriptions into consideration, the performance enormously increases. When *k*=5, the visit-level precision and code-level accuracy of RETAIN improve 37% and 42% respectively. The performance of MLP is better than that of RETAIN, but it is still lower than other RNN variants. This illustrates that with complicated EHR datasets, simply using multilayer perceptrons cannot work well. Though learning medical embeddings of diagnosis codes improves the predictive performance, the accuracy of MLP + is still lower than that of most approaches. This directly validates that applying recurrent neural networks to diagnosis prediction task is reasonable.

For the two location-based attention approaches, RNN _*a*_ and Dipole, the performance is better than that of RNN, which demonstrates that attention mechanisms can help the models to enhance the predictive ability. Comparison between RNN _*a*_ and Dipole confirms that when the size of visit sequences is big, bidirectional recurrent neural networks can remember more useful information and perform better than one directional recurrent neural networks.

Based on all the above analysis, we can safely conclude that learning diagnosis code embeddings with descriptions indeed helps all the state-of-the-art diagnosis prediction approaches to significantly improve the performance on different real world datasets.

## Discussions

The main contribution of this work is to incorporate code descriptions to improve the prediction performance of state-of-the-art models. The experimental results on two real datasets confirm the effective of the proposed framework. Next, we further discuss the performance changes with the degree of data sufficiency and the representations leaned by the proposed framework.

### Data Sufficiency

**Fig. 2:**
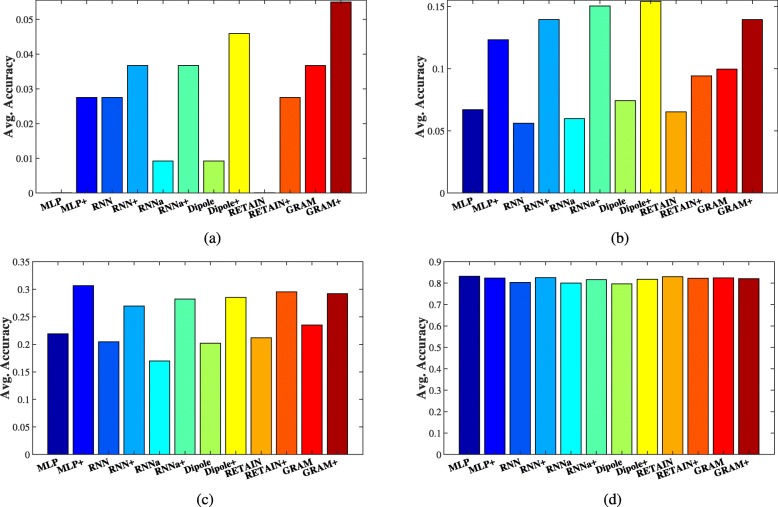
Code-Level Accuracy *@*20 of Diagnosis Prediction on the MIMIC-III Dataset. **a** 0-25. **b** 25-50. **c** 50-75. **d** 75-100

In healthcare, it is hard to collect enough EHR data for those rare diseases. In order to validate the sensitivity of all the diagnosis prediction approaches to data sufficiency, the following experiments are conducted on the MIMIC-III dataset. We first calculate the frequency of category labels appeared in the training data, then rank these labels according to the frequency, and finally divide them into four groups: 0-25, 25-50, 50-75 and 75-100. The category labels in group 0-25 are the most rare ones in the training data, while the labels in group 75-100 are the most common ones. We finally compute the average accuracy of labels in each group. The code-level accuracy *@*20 on the MIMIC-III dataset is shown in Fig. 2. X-axis denotes all the base models and their corresponding enhanced approaches, and Y-axis represents the average accuracy of the approaches.

From Fig. 2, we can observe that the accuracy of all the enhanced diagnosis prediction approaches is higher than that of all the base models in the first three groups. Even though MLP and RETAIN achieve higher accuracy compared with RNN, RNN _*a*_ and Dipole as shown in Table 4, the accuracy of both approaches is 0 in group 0-25. However, when generalizing the proposed framework on MLP and RETAIN, they all make some correct predictions for rare diseases. This observation also can be found in groups 25-50 and 50-70. Therefore, this observation validates that considering the medical meanings of diagnosis codes indeed helps existing models to enhance their predictive ability even without sufficient training EHR data.

In Fig. 2d, all the labels have sufficient and abundant training EHR data. Thus, all the approaches achieve comparable performance. This result again confirms that the enhanced approaches improve the predictive performance on those rare diseases, i.e., the labels without sufficient training EHR records. Among all the base models, GRAM obtains the highest accuracy in groups 0-25, 25-50 and 50-75, which illustrates the effectiveness of incorporating external medical knowledge. Furthermore, learning medical embeddings with ontologies still improves the predictive accuracy, which can be observed from both Fig. 2 and Table 4.

### Interpretable Representation

**Fig. 3:**
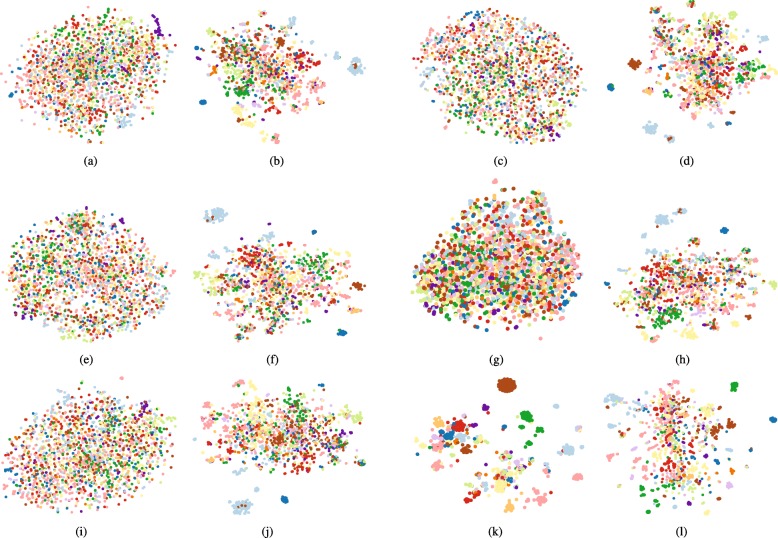
*t*-SNE Scatterplots of Medical Codes Learned by Predictive Models. **a** MLP. **b** MLP +. **c** RNN. **d** RNN +. **e** RNN _*a*_. **f** RNN _*a*_+. **g** Dipole. **h** Dipole +. **i** RETAIN. **j** RETAIN +. **k** GRAM. **l** GRAM +

For diagnosis prediction task, interpreting the learned medical code embeddings is significantly important. Thus, we conduct the following experiments to qualitatively demonstrate the learned representations by all the approaches on the MIMIC-III dataset. W randomly select 2000 diagnosis codes and then plot them on a 2-D space with *t*-SNE [42] shown in Fig. 3. The color of the dots represents the first disease categories in CCS multi-level hierarchy as [3]. We can observe that except GRAM, the remaining baselines cannot learn interpretable representations. However, after considering the semantic meanings learned from diagnosis code descriptions, all the proposed approaches can learn some interpretable cluster structures in the representations. Especially for GRAM +, it not only maintains the advantages of GRAM, but also improves the prediction accuracy. From Fig. 3, we come to a conclusion that the proposed semantic diagnosis prediction framework is effective and interpretable even when the training EHR data are insufficient.

## Conclusions

Diagnosis prediction from EHR data is a challenging yet practical research task in healthcare domain. Most state-of-the-art diagnosis prediction models employ recurrent neural networks to model the sequential patients’ visit records, and exploit attention mechanisms to improve the predictive performance and provide interpretability for the prediction results. However, all the existing models ignore the medical descriptions of diagnosis codes, which are significantly important to diagnosis prediction task, especially when the EHR data are insufficient.

In this paper, we propose a novel and effective diagnosis prediction framework, which takes the medical meanings of diagnosis codes into account when predicting patients’ future visit information. The proposed framework includes two basic components: diagnosis code embedding and predictive model. In the diagnosis code embedding component, medical representations of diagnosis codes are learned from their descriptions with a convolutional neural network on top of pre-trained word embeddings. Based on the learned embeddings, the input visit information is embedded into a visit-level vector representation, which is then fed into the predictive model component. In the predictive model component, all the state-of-the-art diagnosis prediction models are redesigned to significantly improve the predictive performance by considering diagnosis code meanings. Experimental results on two real world medical datasets prove the effectiveness and robustness of the proposed framework for diagnosis prediction task. An experiment is designed to illustrate that the enhanced diagnosis prediction approaches outperform all the corresponding state-of-the-art approaches under insufficient EHR data. Finally, the learned medical code representations are visualized to demonstrate the interpretability of the proposed framework.

## Data Availability

The MIMIC-III dataset can be obtained from the line: https://mimic.physionet.org/gettingstarted/access/.

## Abbreviations

BRNN Bidirectional recurrent neural network; CCS: Clinical classifications software
CNN: Convolutional neural networks
DAG: Directed acyclic graph
Dipole: Attention-based bidirectional recurrent neural networks
Dipole +: Enhanced attention-based bidirectional recurrent neural networks
EHR: Electronic health records
GRAM: Graph-based Attention model
GRAM +: Enhanced graph-based attention model
GRU: Gated recurrent unit
LSTM: Long-short term memory
MIMIC-III: Medical information mart for intensive care
MLP: Multilayer perceptron
MLP +: Enhanced multilayer perceptron
RETAIN: Reverse time attention mechanism
RETAIN +: Enhanced reverse time attention mechanism
RNN: Recurrent neural networks
RNN +: Enhanced recurrent neural network
RNN _*a*_: Attention-based recurrent neural network
RNN _*a*_+: Enhanced attention-based recurrent neural network
SDA: Stacked denoising autoencoders
T-LSTM: Time-aware long-short term memory
